# Pioglitazone retrieves hepatic antioxidant DNA repair in a mice model of high fat diet

**DOI:** 10.1186/1471-2199-9-82

**Published:** 2008-09-26

**Authors:** Pi-Jung Hsiao, Tusty-Jiuan Hsieh, Kung-Kai Kuo, Wei-Wen Hung, Kun-Bow Tsai, Ching-Hsiu Yang, Ming-Lung Yu, Shyi-Jang Shin

**Affiliations:** 1 Division of Endocrinology and Metabolism, Department of Internal Medicine; Kaohsiung Medical University, Kaohsiung, Taiwan; 2 Graduate Institute of Medical Genetics,Kaohsiung Medical University, Kaohsiung, Taiwan; 3 Hepatobiliary Division, Department of Surgery; Kaohsiung Medical University, Kaohsiung, Taiwan; 4 Department of Pathology, Kaohsiung Medical University, Kaohsiung, Taiwan; 5 Hepatobiliary Division, Department of Internal Medicine, Kaohsiung Medical University, Kaohsiung, Taiwan

## Abstract

**Background:**

Pioglitazone was reported to improve hepatic steatosis and necroinflammation in human studies. To investigate whether the hepato-protective effect of pioglitazone was associated with an improvement of antioxidant defense mechanism, oxidative DNA damage and repair activity were determined in a high fat diet model. Male C57BL/6 mice were respectively fed with a 30% fat diet, the same diet with pioglitazone 100 mg/kg/day, or a chow diet as control for 8 weeks. Tissue oxidative stress was indicated by malondialdehyde concentration. Oxidative DNA damage was detected by immunohistochemical 8-oxoG staining. Enzymatic antioxidant defense was detected by the real-time PCR of superoxide dismutase (*Sod1, Sod2*) and DNA glycosylase (*Ogg1, MutY*). Oxidative DNA repair was detected by immunohistochemical staining and western blotting of OGG1 expression.

**Results:**

Our results show that hepatic steatosis was induced by a high-fat diet and improved by adding pioglitazone. Malondialdehyde concentration and 8-oxoG staining were strongly increased in the high-fat diet group, but attenuated by pioglitazone. Gene expressions of antioxidant defense mechanism: *Sod1, Sod2, Ogg1 *and *MutY *significantly decreased in the high-fat diet group but reversed by pioglitazone co-administration.

**Conclusion:**

The attenuation of hepatic oxidative DNA damage by pioglitazone in a high-fat diet may be mediated by up-regulation of the antioxidant defense mechanism and oxidative DNA repair activity. The diminution of oxidative damage may explain the clinical benefit of pioglitazone treatment in patients with non-alcoholic fatty liver disease.

## Background

The prevalence of non-alcoholic fatty liver disease (NAFLD) in the general population is estimated to be between 14–24% in wealthy countries. NAFLD presents as a hepatic manifestation of metabolic syndrome and is characterized by triglyceride accumulation, lobular necroinflammation, and may progress to fibrosis, cirrhosis and hepatocellular carcinoma. Recently, NAFLD has been speculated to be initiated by insulin resistance [1,2]. Some investigations have demonstrated that reactive oxygen species (ROS) production increases during insulin resistance and then triggers lipid peroxidation, mitochondrial dysfunction and releasing of several cytokines, such as tumor necrosis factor-α (TNF-α) [3,4]. In human studies, it has been shown that a high-fat diet increases the hepatocellular free fatty acid pool and increases ROS formation in mitochondria. Furthermore, ROS directly damage mitochondrial DNA and trigger the progression of simple steatosis to steatohepatitis, fibrosis, cirrhosis and possible formation of hepatocelluar carcinoma [3-6].

Among various threats to living cells, ROS injury to DNA is as a result of cellular respiration, metabolism, and environmental insults. Damaged mitochondria are the major source of ROS production and go into a vicious cycle of increasing DNA damage by the ROS. ROS can damage cellular DNA directly by causing strand breaks and base oxidation in DNA [7,8]. Physiologically, defense mechanism against oxidative stress includes antioxidant defense and repair mechanism. Superoxide dismutases (SOD1 and SOD2) represent a family of enzymatic antioxidant defense involved in converting superoxide into peroxide. *Sod1 *encodes cytosolic SOD and *Sod2 *encodes mitochondrial SOD, which is more important antioxidant defense than *Sod1 *to prevent ROS production in the mitochondria [9,10]. Mitochondrial DNA is more prone to oxidative damage than nuclear DNA. The most common oxidation product of DNA, 7,8-dihydro-8-oxoguanine (8-oxoG), is particularly abundant and stable with approximately ~180 guanines oxidized to 8-oxoG per mammalian cell per day. On the contrary, base excision repair (BER) is the major repair system allowing mitochondria to efficiently repair oxidized DNA bases such as 8-oxoG. BER, initiated by 8-oxoG glycosylase (OGG1) and cooperated by MutY homolog DNA glycosylase (MutY), is the most important defense against 8-oxoG mutation in mitochondria [11] The *Ogg1 *gene encodes DNA 8-oxoG glycosylase which recognizes and removes 8-oxoG from DNA. MutY homolog DNA glycosylase (MutY) is another monofunctional glycosylase, which specifically excises adenine misincoporated opposite 8-oxoG in the mismatch repair (MMR) system against oxidative stress. Evidence has shown OGG1 and MutY form a cooperative defense against G: C to T: A transversions [11-14]. Thus, *Ogg1 *and *MutY *gene expression represents oxidative repair activity of DNA.

Pioglitazone, a derivative of thiazolidinedione (TZD), is a peroxisome proliferator-activated receptor-γ (PPARγ) agonist that is used to treat type 2 diabetes by increasing insulin sensitivity. In subjects with NAFLD, insulin resistance is associated with hyperinsulinemia, hyperglycemia, high plasma free fatty acid, and low plasma adiponectin levels. TZDs may reverse these abnormalities in NAFLD subjects [3,5,15,16]. More recently, a placebo-controlled trial in subjects with nonalcoholic steatohepatitis (NASH) showed significant improvement of histological features occurred in subjects receiving pioglitazone as compared with placebo group [17]. Reduction of the insulin resistance is generally thought to be the mechanism of TZD treatment in NAFLD [2]. In addition, oxidative stress has been demonstrated as a causal role in insulin resistance and TZDs is reported to reduce vascular oxidative stress [18]. Therefore, it is intriguing to identify whether the hepato-protective mechanism of TZDs is mediated by attenuating the hepatic oxidative DNA damage. In a mice model of high fat diet, we investigated the changes of oxidative stress, indicated by malondialdehyde (MDA) and oxidative DNA damage, indicated by 8-oxoG. We further explored up-regulation of the antioxidant defense and repair genes (SOD1, SOD2, OGG1 and MutY) by pioglitazone and investigated the improvement of hepatic steatosis by reversal of the oxidative stress and damage.

## Methods

Male C57BL/6 inbred mice, aged 8 weeks, were obtained from BioLASCO Technology (Charles River Taiwan Ltd). All mice received standard animal care under the supervision of our Institutional Animal Care and Use Committee. The mice were caged in an air-conditioned animal facility at 23°C on a 12-h light: dark cycle and were maintained with free access to water and food. All the mice were fed with standard chow diet (Basal diet™ 5755, PMI Nutrition International, St. Louis, MO, USA) for one week. The composition of this basal chow diet was 60.6% (wt/wt) carbohydrate (starch 43.6% and sucrose 16.9%), 10% fat, 19% protein, 4.3% fiber, 5% mineral mixture and 0.2% vitamin mixture. They were then divided into three groups: (1) chow diet (n = 5); (2) high-fat diet (30%) (n = 5) (catalog #7166, PMI Nutrition International, Saint Louis, MO, USA); (3) high-fat diet and gastric gavage with pioglitazone 100 mg/kg/day (n = 5). The high-fat diet, based on basal diet 5755 (contained 40.6% carbohydrate (dextrin 23.6% and sucrose 15%), 15% corn oil, 15% lard, 19% protein, 4.3% fiber, 5% mineral mixture and 0.2% vitamin mixture) provided 53.1% of calories from corn oil and lard. Pioglitazone was kindly provided by Takeda Chemical Industries (Taiwan), Ltd. Animals were fed in these groups for 8 weeks prior to euthanasia.

### Biochemical analysis

Throughout the experiment, body weight was recorded daily. At the end of experiment, the animals were sacrificed after an overnight fast. They were euthanized by intraperitoneal injection of the anaesthetic Zoletil (10 mg/kg) (Virbac, Carros, France). Blood samples were collected from the heart at the time of sacrifice for measurement of plasma glucose, serum cholesterol, triglyceride and alanine aminotransferase. These parameters were assessed using an autoanalyser (Roche Diagnostics, Taipei, Taiwan). After rinsing with phosphate buffered saline (PBS), the livers were immediately cut into pieces on ice. Two pieces of liver were fresh-frozen in liquid nitrogen for storage at -80°C for measurement of hepatic triglyceride content and subsequent RNA isolation. Hepatic lipids were extracted from tissue with chloroform/methanol (2:1, vol/vol) and centrifugation. Then, the supernant was subsequently analyzed for TG content by colorimetric enzymatic hydrolysis (Triglyceride GPO-Triginder reagent; Sigma, St. Louis, MO). The remaining liver samples, fixed with 4% paraformaldehyde and embedded in paraffin, were prepared for morphological and immunohistochemical analyses. Thiobarbituric acid reactive substances (TBARS) assay was used to measure the tissue concentration of malondialdehyde (MDA), which is a product of lipid peroxidation, as an indicator of oxidative stress (ZeptoMetrix, Co, NewYork).

### Histologic and immunohistochemical analysis

The dissected liver specimens were fresh-frozen and fixed in Tissue-Tek^R ^O.C.T compound (Sakura Finetechnical Co., Tokyo, Japan) for oil-red O staining. Other parts of the dissected specimens were embedded in paraffin for hematoxylin and eosin (H&E) and Masson trichrome staining. Some sections of liver were processed and incubated with a mouse primary monoclonal antibody for 8-hydroxyguanosine (diluted 1:2000 in PBS, pH 7.4) or rabbit anti-mouse OGG1 (1: 2000 in PBS, pH 7.5) (Alpha Diagnostic Intl. Inc., San Antonio, TX, USA) overnight at 4°C. The pretreated slides were stained with reagents from a commercially available kit applying a streptavidin-HRP method with 3, 3-diaminobenzamide (DAB) as a chromogen (DAKO Corporation, Carpinteria, CA, USA). Normal mouse serum was substituted for the primary antibody as negative control. Then, the slides were counterstained with hematoxylin and examined under light microscopy. The histologic scoring system validated by the Pathology Committee of the NASH Clinical Research Network for human was applied for semi-quantitative evaluation of these liver specimens of mice. The histologic features of activity score included sum of steatosis (0–3), lobular inflammation (0–2), hepatocellular ballooning (0–2) and fibrosis (0–4) [19]. The immunostaining intensity of 8-oxoG and OGG1 were graded according to the following score: "0"= no staining, "1" = weak staining, "2"= moderate staining, "3"= strong staining. The histologic grading and intensity of immunostaining were evaluated of 10 fields in high magnification (400×) of each group by one hepatologist and one pathologist who had no idea of the sources of the sections.

### Real-time PCR (RT-PCR) for genes related to oxidative defense mechansim

Total hepatic RNA was extracted using Trizol reagent according to the supplier's protocol, with absorbancy measured at 260 nm. Briefly, 1 μg of total RNA was used to synthesize first-strand cDNAs with an iScript™ cDNA Synthesis Kit (Bio-Rad Laboratories, INC., CA, USA). Then, the first-strand cDNA was diluted with water in a ratio of 1:9, and the aliquots were processed to amplify the genes related to oxidative defense mechanism (*Sod1, Sod2, Ogg1 *and *MutY*) and β-actin cDNA fragments with iQ™ SYBR Green Supermix (Bio-Rad Laboratories, INC., CA, USA). RT-*PCR *was performed in a Bio-Rad MiniOpticon Real-Time PCR System (Bio-Rad Laboratories, INC., CA, USA). The primers used for RT-PCR were purchased from Bio Basic Inc. (Ontario, Canada). The primer sequences were listed in table 1.

**Table 1:** Primer sequences of genes involved in antioxidant defense and DNA repair

Gene	Sequence	Gene ID number
*Sod1*	F: 5'-gcggtgaaccagttgtgttgtc-3'	NM_011434
	R: 5'-cagtcacattgcccaggtctcc-3'	
*Sod 2*	F: 5'-atgttacaactcaggtcgctcttc-3'	NM_013671
	R: 5'-tgatagcctccagcaactctcc-3'	
*Ogg1*	F: 5'-gtgactacggctggcatcc-3'	NM_010957
	R: 5'-aggcttggttggcgaagg-3'	
*MutY*	F: 5'-cattgcttccatcgcctttgac-3'	NM_133250
	R: 5'-gctaagttccagaggtgatgagag-3'	
*Beta-actin*	F: 5'-gaaatcgtgcgtgacatc-3'	NM_007393
	R: 5'-ccatacccaagaaggaagg-3'	

*Ogg1*: codes for oxoguanine DNA glycosylase

*MutY*: codes for MutY homolog DNA (A/G specific adenine) glycosylase

*Sod1*, codes for cytosolic superoxide dismutase; *Sod2*, codes for mitochondrial superoxide dismutase.

*Beta-actin *as a competitive control

### Western blot analysis

OGG1 expression in liver was determined with Western blot analysis. Thirty micrograms of protein was separated on a 10% SDS-polyacrylamide gel running with constant current for 2.5 hrs. After electrophoresis, the proteins were transferred to PVDF membrane (Millipore, Bedford, M.A, USA), assembled in a Bio-Rad Transblot and immersed in blocking buffer overnight at 4°C. Then, the membrane was used to detect the levels of mouse OGG1 protein with a primary antibody against mouse OGG1 (Alpha Diagnostics Intl. Inc., San Antonio, TX, USA) or β-actin (Alpha Diagnostics Intl. Inc., San Antonio, TX, USA) as a control. Immune complexes were visualized using ECL plus detection reagents (Amersham International, NJ, USA). Quantitative comparison of the fluorescent images was achieved with a densitometer.

### Statistical analysis

All statistical analyses were performed using SPSS 10.0 for Windows (SPSS Inc., Chicago, IL, USA). Values are presented as mean ± S.E. Statistical significance was determined as *p *< 0.05 using non-parametric Kruskal-Wallis test among three groups or Wilcoxon rank-sum tests between two groups.

## Results

In this study, biochemical data of three groups are shown in table 2. Body weight gain increased significantly in group of high fat diet plus pioglitazone than groups of chow diet or high fat diet. Fasting blood glucose, serum cholesterol and hepatic triglyceride content were significantly different among three groups, especially higher in groups of high fat diet than the other two groups. Tissue oxidative stress, indicated by MDA concentration, was significantly higher in high fat diet group and reversed markedly by co-administration with pioglitazone.

**Table 2:** Biochemical data of three groups

Group	Chow diet (n = 5)	High fat diet (n = 5)	High fat diet + PioG (n = 5)	*P*
Initial BW (gm)	21.1 ± 0.3	21.6 ± 0.4	20.8 ± 0.5	0.538
Final BW (gm)	24.8 ± 0.9	26.0 ± 0.9	27.6 ± 0.7	0.088
BW gain (gm)	3.7 ± 0.7	4.4 ± 0.8	5.0 ± 0.5	0.025
Blood glucose (mg/dl)	125.0 ± 8.0	169.2 ± 4.2	147.6 ± 7.2	0.011
Cholesterol (mg/dl)	59.5 ± 10.4	159.7 ± 37.1	151.6 ± 14.2	0.008
Triglyceride (mg/dl)	71.9 ± 17.1	102.8 ± 27.6	95.9 ± 13.0	0.355
ALT (IU/L)	14.8 ± 5.5	13.0 ± 5.9	27.7 ± 13.6	0.691
Hepatic TG (mg/g protein)	72.0 ± 19.9	177.5 ± 61.4	153.6 ± 60.9	0.023
MDA (nmol/mg protein)	5.9 ± 1.0	9.7 ± 0.8	5.5 ± 0.3	0.008

Values are mean ± S.E.

ALT: alanine aminotransferase; Hepatic TG: hepatic triglyceride content; MDA: malondialdehyde

Kruskal-Wallis test were used for statistical analysis; *p *< 0.05 was indicated significant.

**Figure 1:**
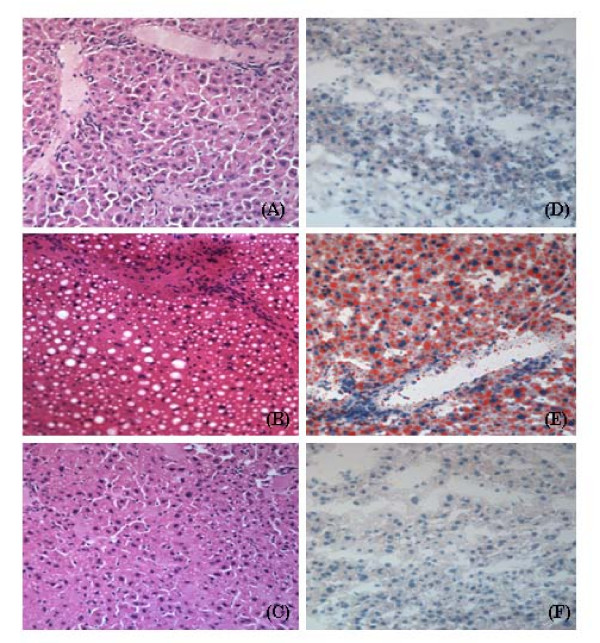
**Histopathology of HE stained liver sections (400×) in (A) chow diet; (B) high-fat diet; (C) high-fat diet co-administered with pioglitazone.** Histopathology of oil-red O stained liver sections (200×) in (D) chow diet; (E) high-fat diet; (F) high-fat diet co-administered with pioglitazone. Hepatic steatosis is strongly induced by high fat diet and improved by co-administration of pioglitazone.

As compared to control mice (A and D in Figure 1), the hematoxylin-eosin staining and oil-red O staining showed larger and more lipid droplet accumulation in liver parenchyma in high-fat diet group (B and E in Figure 1, respectively). Hepatic steatosis was improved by pioglitazone co-administration (C and F in Figure 1, respectively) with smaller lipid droplets than those of high fat group. The activity score of the histopathologic grading, shown in chow diet (3.75 ± 0.96), high fat diet (6.0 ± 0.82) and high fat diet adding pioglitazone (4.0 ± 1.91), was significantly different among three groups (*p *< 0.05, Kruskal-Wallis test). There was no obvious fibrosis identified by Masson trichrome stain among three groups (not shown). However, the improvement of steatosis, necroinflammation and liver cell ballooning by adding pioglitazone was also confirmed by the semi-quantitative scoring system.

**Figure 2:**
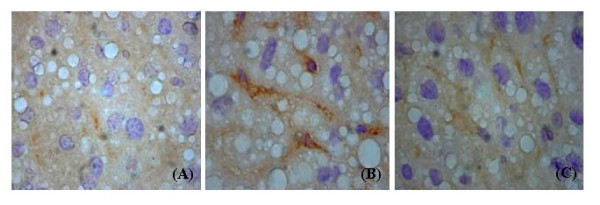
I**mmunohistochemistry of hepatic 8-oxoG expression.** (A) chow diet (400×); (B) markedly increased 8-oxoG expression in hepatocytes of high fat diet (400×); (C) attenuation of the increase of 8-oxoG expression by pioglitazone administration with high-fat diet (400×).

Oxidative DNA damage was determined with 8-oxoG staining (Figure 2). The intensity of staining was faint (score 0.6 ± 0.13) in chow diet group and much stronger (score 2.81 ± 0.09) in high fat diet group. In comparison with that of chow diet (Figure 2A), it demonstrated a noticeable increase of 8-oxoG in hepatocytes (Figure 2B). Pioglitazone administration in the high-fat group reduced the intensity of 8-oxoG staining (score 1.32 ± 0.10) in liver (Figure 2C). The intensity of 8-oxoG staining showed significant difference among three groups (*p *< 0.001, Kruskal-Wallis test).

**Figure 3:**
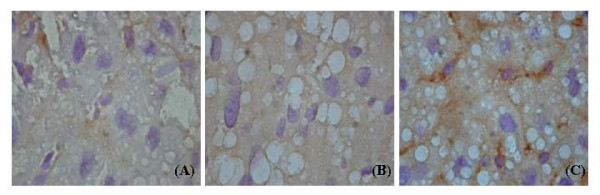
**Immunohistochemistry of hepatic OGG1 expression.** (A) basal OGG1 activity in chow diet (400×); (B) decreased OGG1 activity in high-fat diet (400×); (C) reversal of decreased OGG1 expression in hepatocytes co-administered high fat diet and pioglitazone (400×).

The immunohistochemical staining of OGG1 expression was less apparent in the high-fat diet (staining score 0.19 ± 0.09, Fig. 3B) than in chow diet group (staining score 0.67 ± 0.13, Figure 3A). Mice treated with pioglitazone increased expression of OGG1 in hepatocytes (staining score 2.77 ± 0.09, Figure 3C). Intensity of the OGG1 staining expressed faint in high fat diet, however, it was significantly enhanced by adding pioglitazone (*p *< 0.001, Kruskal-Wallis test).

**Figure 4:**
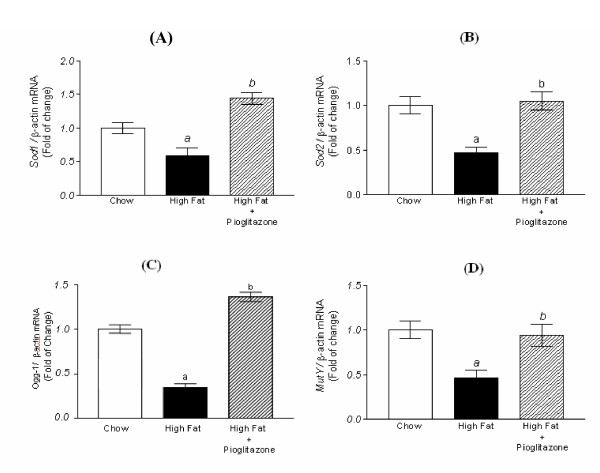
**Real-time PCR for mRNA expression of genes related to enzymatic antioxidant defense and DNA repair, normalized by beta-actin.** (A) *Sod1*. (B) *Sod2*. (C) *Ogg1*. (D) *MutY*. Each experiment was performed in duplicate and is represented as the mean ± S.E of five mice in each group. (^a ^*p *< 0.01 compared with chow diet; ^b ^*p *< 0.01 compared with high fat diet by Wilcoxon rank-sum test).

**Figure 5:**
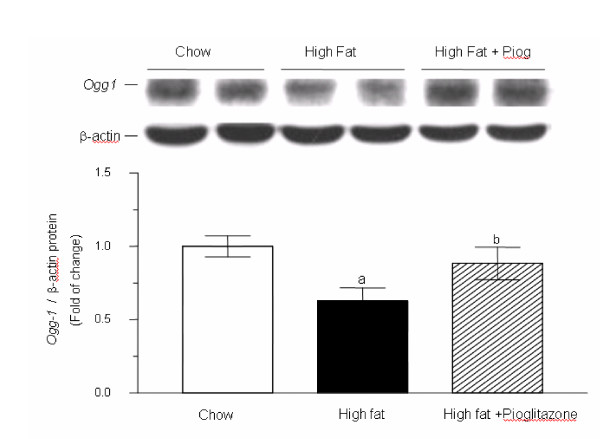
**Western blotting of hepatic OGG1 expression. **There was a significant decrease in OGG1 expression in the high-fat diet compared with chow diet (^a ^*p *< 0.01, Wilcoxon rank-sum test) but co-administered of pioglitazone with the high-fat diet significantly reversed the decreased OGG1 expression in comparison with that of the high-fat diet alone (^b ^*p *< 0.01, Wilcoxon rank-sum test). Each experiment was performed in duplicate and is represented as the mean ± S.E of five mice in each group.

Gene expressions of *Sod1, Sod2, Ogg1 *and *MutY *mRNA, involved in enzymatic antioxidant defense and oxidative DNA repair, demonstrated a parallel trend of down-regulation in high fat diet but significant up-regulation by adding pioglitazone (Figure 4). The mRNA expression of *Ogg1 *in high-fat group declined significantly to 34% of that in chow diet group. However, it was significantly up-regulated to 1.36-fold of the chow diet group and 4-times of the high-fat diet group by co-administration of pioglitazone. Western blotting analysis for OGG1 demonstrated a similar trend with the expression of OGG1 mRNA. The OGG1 protein expression revealed a substantial decrease in high fat diet and retrieved significantly by pioglitazone co-administration compared with the high fat diet group alone (Figure 5).

## Discussion

Our study clearly demonstrated that oxidative DNA damage is increased in a steatotic liver of mice fed with a high fat diet. The oxidative DNA damage induced by a high fat diet can be attenuated by the pioglitazone treatment. Our findings include: (1) Tissue oxidative stress was significantly enhanced with higher MDA content and stronger 8-oxoG staining in high fat diet, but attenuated by co-administration of pioglitazone. (2) Gene expression of enzymatic antioxidant defense and DNA repair mechanism, including *Sod1, Sod2, Ogg1 *and *MutY*, all were down-regulated in high fat diet but significantly reversed by adding pioglitazone in high fat diet.

High-fat diet and lack of exercise are causing a burdensome epidemic of obesity, diabetes, and NAFLD. NAFLD is characterized by triglyceride accumulation in hepatocytes, which probably develop steatohepatitis, fibrosis, and even hepatocellular carcinoma formation by secondary hit. Although the mechanisms responsible for NAFLD are still unclear, it may be initiated by insulin resistance. It has been reported that insulin resistance will enhance ROS production and oxidative stress. Then, the oxidative stress accelerates the progression of NAFLD to steatohepatitis through a series of signaling cascade pathways, such as mitogen-activated protein kinases and nuclear factor κB [1-3,5,20]. ROS production is significantly increased in subjects with nonalcoholic steatohepatitis and in rats fed with high fat diet [21-23]. Chronic oxidative stress secondary to ROS overproduction and antioxidant deletion is linked to chemically altering DNA, protein and lipids [3,4,20,22,24]. In this study, we demonstrated that increased oxidative damage with more 8-oxo-G accumulation existed in steatotic hepatocytes in mice fed a high-fat diet. Additionally, genes expression for enzymatic antioxidant defense and DNA repair mechanism (*Sod1, Sod2, Ogg1 *and *MutY *mRNA) apparently decreased in the livers of mice fed with high fat diet. These results apparently prove that hepatic oxidative DNA damage was markedly raised in accompany with diminished antioxidant defense and repair activity in a mice model of high fat diet. This preliminary finding implicate that high fat intake may result in hepatic steatosis as well as accumulation of oxidative DNA damage. And the depletion of antioxidant defense and impairment of DNA repair mechanism may contribute to the progression of NAFLD.

Normally, PPARγ is predominantly expressed in adipose tissue and expressed at very low levels in liver. However, in animal models of insulin resistance and fatty livers, the hepatic expression of PPARγ is markedly increased. Possibly, PPARγ activation is strongly involved in the development of NAFLD [5,15,25-27]. Human studies have demonstrated that TZD can reverse many abnormalities in nonalcoholic steatohepatitis. The histological features of steatosis, ballooning necrosis and centrilobular inflammation significantly improved in subjects with nonalcoholic steatohepatitis receiving pioglitazone treatment compared with placebo [17,28]. Several mechanisms have been explored to explain the hepato-protective effects of TZDs in the treatment of NAFLD, including: amelioration of insulin resistance, increasing adiponectin concentration, reducing the TNF-α production, activation of AMP-related protein kinase and inactivation of the intracellular pro-inflammatory signaling pathway [4-6,29]. In an animal study, pioglitazone treatment increased the Cu, Zn-SOD activity, decreased catalase activity and the level of peroxidation products of liver and kidney in diabetic rabbit or rats induced by alloxan [30,31]. Evidence has demonstrated that increased oxidative stress has recently been recognized as an essential role in insulin resistance [18]. In this study, we demonstrated that MDA and 8-oxo-G expression markedly increased with reciprocal decrease of *Sod1, Sod2, Ogg1 *and *MutY *mRNA and OGG1 protein expression in liver tissue of high fat diet. As well, the above changes are reversed by adding pioglitazone. To our knowledge, this is the first study to demonstrate that pioglitazone treatment, by enhancing enzymatic antioxidant defense and DNA repair mechanism, could attenuate the hepatic oxidative stress and DNA damage induced by high fat diet. It may indicate one of the hepato-protective mechanisms of pioglitazone is mediated by retrieving oxidative DNA repair, which in turn block the vicious cycle of ROS production, improve insulin sensitivity and halt the pro-inflammatory signaling transduction. However, is the hepato-protective effect of pioglitazone due to the improvement of insulin sensitivity, and/or due to a direct effect of DNA repair on liver cells? This in vivo study of dietetic model of hepatic steatosis and co-administration with pioglitazone is limited to conclude.

In the progression of NAFLD to steatohepatitis, cirrhosis and carcinoma, ROS plays an essential role to trigger lipid peroxidation, induce mitochondrial dysfunction, stimulate cytokine release, and activate stellate cell. There is growing evidence to show mitochondrial dysfunction associated with ROS overproduction participate a vicious cycle leading to worsening of NAFLD [3-5]. If the excess ROS persists, the risk of continuous oxidative DNA damage, genetic mutation and the cancer formation increases [7-10]. In mammalian cells, there are at least two defense pathways involved to overcome the oxidative damage from ROS. They are enzymatic antioxidant defenses (such as SOD, catalase) and DNA repair mechanisms (OGG1 and MutY). The vast majority of small oxidative DNA damages, especially in mitochondria, are repaired by the BER system. BER primarily repairs the mutation caused by ROS generation during internal metabolism to keep genome integrity. OGG1 is the only glycosylase for 8-oxoG removal in mouse mitochondria and plays more important role in mitochondria than nuclear DNA repair [9,11-14]. Our result demonstrated the insulin sensitizer, pioglitazone, is capable to enhance the mitochondrial antioxidant defense (*Sod2*) and DNA repair (*Ogg1 and MutY*) system to attenuate oxidative stress and oxidative DNA damage (cytosol 8-oxoG). This finding is consistent with the hepatic salvage effect of pioglitazone in the human studies reported by Belfort et al and Lutchman et al [17,28]. Our result provided evidence that retrieval of mitochondrial antioxidant defense mechanism by pioglitazone may explain another therapeutic mechanism of TZD for NAFLD.

## Conclusion

Our study clearly demonstrated that there was significantly increased oxidative stress and oxidative DNA damage in steatotic livers of a mice model fed with a high fat diet. The oxidative DNA damage induced by a high fat diet can be attenuated by pioglitazone treatment through up-regulation of the antioxidant defense and oxidative DNA repair genes (*Sod1, Sod2, Ogg1 *and *MutY*).

## Abbreviations

NAFLD: nonalcoholic fatty liver disease; ROS: reactive oxygen species; TNF-α: tumor necrosis factor-α; SOD: superoxide dismutase; 8-oxoG: 7,8-dihydro-8-oxoguanine; BER: base excision repair; OGG1: 8-oxoG glycosylase; MutY: MutY homolog DNA glycosylase; PPARγ: proliferator-acitvated receptor-γ; TZD: thiazolidinedione; NASH: nonalcoholic steatohepatitis; MDA: malondialdehyde; TBARS: Thiobarbituric acid reactive substances assay.

## Authors' contributions

P-JH, T-JH, K-KK: conception and design, acquisition of data, analysis and interpretation of data, drafting manuscript. W-WH, K-BT, C-HY, M-LY: acquisition, analysis and interpretation of data. S-JS: drafting and revising manuscript, final approval of the version for submission.
